# Mastery learning in a bachelor’s of nursing program: the Roseman University of Health Sciences experience

**DOI:** 10.1186/s12912-019-0371-x

**Published:** 2019-11-06

**Authors:** Martin S. Lipsky, Catherine J. Cone, Susan Watson, Phillip T. Lawrence, May Nawal Lutfiyya

**Affiliations:** 1 Office of the Chancellor, Roseman University of Health Sciences, 10920 S River Front Parkway, South Jordan, Utah USA; 2 College of Pharmacy, Roseman University of Health Sciences, 10920 S River Front Parkway, South Jordan, Utah USA; 3 College of Nursing, Roseman University of Health Sciences, 10920 S. River Front Parkway, South Jordan, Utah USA; 4 College of Dental Medicine, Roseman University of Health Sciences, 10920 S. River Front Parkway, South Jordan, Utah USA

**Keywords:** Nursing mastery learning, compentency based education, deliberative practice, feedback

## Abstract

**Background:**

Roseman University of Health Sciences (RUHS) developed and delivers a mastery learning curriculum designed for students to acquire the knowledge and skills to become competent nurses. Despite a trend in nursing education to adopt competency-based education (CBE) models, there is little in the nursing literature about programs based on a mastery model. The aim of this study is to describe an undergraduate nursing program built on a mastery learning model and to report on program outcome measures.

**Methods:**

The 18-month BSN nursing program is divided into blocks, varying in length and focusing on a single subject. Students must demonstrate mastery, defined as ≥90% on an assessment, to pass a block. Recognizing the critical nature of health care, educators seek methods to assure that practitioners become competent to perform the services they provide.

Program outcomes reported include comparisons to national standards and RUHS student exit survey data.

**Results:**

From 2013 to 2017 the RUHS College of Nursing students’ pass rates ranged from 82 to 97% for the National Council Licensure Examination exam compared to national pass rates between 81.8–84.5% during the same time frame. The program completion rate ranged from 86 to 100% and employment rates exceeded accreditation standards. Students reported overall satisfaction with their education as 4.38 and with the block system as 4.74 (5 point Likert scale).

**Conclusions:**

Roseman University’s mastery learning model appears successful as measured by high levels of student satisfaction, outcomes on exams, and degree completion when compared to national averages. The results suggest that other nursing and health profession’s programs can develop a successful mastery based learning model.

## Background

Health professionals affect the health and well-being of the populations they serve. Recognizing the critical nature of health care, educators seek methods to assure that practitioners become competent to perform the services they provide [1]. Several definitions of competency exist. For nursing, Anima and McCoy [2] define competency as acquiring the integration of knowledge, skills, values and attitudes expected and required of a competent nurse.

Mastery learning is gaining acceptance as a method for graduating competent health professionals [3]. Often used synonymously with competency-based, outcome- based, performance-based, and standard-based education, [4] the common denominator among these terms is that learners must demonstrate mastery of a required set of outcomes [5]. Mastery models differ from traditional curricula by defining progression as achieving a series of competencies rather than by an accumulation of credit hours [6]. Despite a trend in nursing education to adopt competency-based education (CBE) models, there is little in the nursing literature about programs based on a mastery model [7].

This paper reviews key features of mastery learning and describes the Roseman University of Health Sciences (RUHS) College of Nursing (CON) 18-month, year-round BSN mastery-based learning model and reports on program outcome measures such as program completion rates and pass rates for licensing exams. In addition, student exit surveys were reviewed to evaluate student assessment of the program. As competency-based healthcare education continues to become more common, RUHS’s CON experience should provide insight to those either contemplating CBE or using a CBE model.

### Mastery Learning Background and Rationale

As conceptualized by Bloom, mastery learning [8] purports that given sufficient time and resources, all students can learn and that aptitude is a measure of the time needed to master content [9]. Based on this assumption, Bloom proposed that instead of a fixed time-based unit educational model, students should demonstrate mastery against a predetermined set of standards before advancing on to the next subject unit [10].

Even though CBE is more than 40 years old, only recently have health science schools started to embrace it [4]. Traditionally most nursing education is subject-centered, time-based, and relies on summative evaluation with little feedback [11, 12]. In contrast, mastery learning features the use of frequent formative assessments to provide feedback and to evaluate whether students have mastered an instructional standard [13]. Mastery models engage learners in deliberative practice with repetitive tasks of increasing difficulty while providing coaching to guide their progress [5].

For students failing to attain mastery, assessment serves as a diagnostic tool to develop an individualized learning plan to guide corrective action and to address deficiencies. Students who initially fail to demonstrate mastery, receive additional opportunities to study and to retest until they achieve mastery. Using a mastery model offers the potential for greater accountability, flexibility and focus on the learner [14].

Setting uniform standards also minimizes learner variation since teaching continues until a predetermined, desired standard is met. This differs from traditional educational models where student achievement is often based on a bell-shaped curve. In contrast, a mastery learning curriculum sets passing at the level of competence needed for proficiency, requiring students to achieve a mastery level score such as 90% compared to the traditional 70% [15] reducing the risk for gaps in knowledge. A meta- analysis across multiple settings found positive effects for mastery learning in 93% of 108 studies with the greatest impact found among weaker students [15]. In the health professions, Cook, et al. [16] found positive associations of mastery learning with skill acquisition and patient outcomes.

### Elements of Mastery Learning and Competency

Key elements of the mastery model include: assuring that students understand expectations, aligning instruction to objectives, providing students with opportunities to practice and to engage in activities that reinforce objectives, and identifying areas that need improvement. In the health professions, mastery learning also emphasizes problem-solving and application of principles and analytic skills [17].

A successful mastery model requires a robust assessment process. Holmboe et. al. [18] recommend that to be effective, assessment requires frequent feedback tied to desired outcomes. Assessments should also be valid, reliable, incorporate both qualitative and quantitative measures and actively involve trainees in the process.

Applying mastery learning to health care education requires broad steps including identifying core competencies identifying content relevant to those competencies, and developing program curriculum tied to assessment planning. The RUHS CON based its core competencies on the American Association of Colleges of Nursing (AACN) guidelines [19] combined with faculty input and oversight. The model required faculty to digest the complex role of a nurse into smaller measurable bite-sized competencies and develop robust assessment methods for each desired outcome.

Simulation, direct observation, and assessment of clinical encounters are essential components used to augment knowledge testing. Other helpful tools include log books, mini-clinical evaluation exercises, and incorporating feedback from multiple sources. To close the loop, a CBE program should continually evaluate its outcomes. One recent study demonstrated that nursing students on medical-surgery rotation using a CBE model performed better than students enrolled in a traditional course (see Table 1) [20].

**Table 1:** Summary of Mastery Model

Mastery Model Features*	
• Small bite-sized units of instruction	
• Clear learning objectives	
• Using multiple educational activities focused on learning objectives (e.g., readings, flipped classroom, deliberative practice, data interpretation)	
• Frequent assessment against objectives	
• Real-time feedback	
• Deliberative practice	
• A set minimum passing standard for each unit to demonstrate mastery	
• Practice or study until mastery standards are met before advancing to the next unit	

*Adapted from Cohen et al. [16]

Despite its proposed benefits, the pros-and-cons of a competency-based model remain hotly debated [21]. While many programs use elements of CBE, RUHS’s CON began with the University leadership’s vision that mastery learning is the best method for educating health professionals and built its entire program on a mastery learning model.

## Methods: The RUHS Model

RUHS is a private, nonprofit health sciences university with campuses in Nevada and Utah. RUHS offers degrees in nursing (BSN), dentistry, pharmacy, and a master’s in business administration as a dual degree with pharmacy and dentistry. All programs are fully accredited and employ a mastery-based model, using criteria and curriculum relevant to their discipline. While other universities utilize CBE, RUHS is unique in building all its educational programs on a mastery learning model.

The CON’s BSN program began in 2006 at its Nevada campus and currently annually enrolls approximately 100 students between its Nevada and Utah campuses. In 2015, the nursing programs on the two campuses merged into a single program that shares the same curriculum, objectives, and assessments. The CON offers both a hybrid program (accelerated bachelor of nursing or ABSN) that incorporates online and face to face education and a more traditional BSN program with in person didactic and clinical rotations. In the US, a BSN or Bachelor of Science in Nursing is a degree level program that typically requires four years of training after completing high school. Successful completion of a degree program includes both didactic and clinical experiences and allows a graduate the opportunity to sit for the nursing licensure examination. Students accepted to Roseman must have either a bachelor’s degree or a minimum of 54 credit hours. All students must have completed prerequisite courses including courses in anatomy and physiology, chemistry, microbiology and statistics prior to admission.

RUHS CON admission requirements include a minimum college grade point average (GPA) of 2.75 and completion of all college prerequisite courses with a grade of C or better. In the US most colleges report a GPA on 4.0 scale where a 4 indicates an A or “outstanding,” a 3.0 a B or “good,” a 2.0 a C or “average,” a D “poor,” and an F “failure.” A GPA is calculated by taking the course grade, multiplying it by the credit value for the course and dividing it by the total credit hours completed in the defined period of study. Other admission requirements include official Test of Essential Academic Skills (TEAS) scores of ≥ 58.7, an interview, and a writing sample. The TEAS is comprised of four content sections: English/language usage, reading, science, and mathematics and is a standardized test used by many nursing schools to predict a candidate’s likelihood for academic success. TEAS scores range from 0 to 100% [22] with a national average about 65-75%. Average cohort GPAs for matriculated RUHS students ranged from 3.2 to 3.3 while average TEAS scores ranged between 74.8 and 83. The students are predominantly female (84%) with an average age of 30.1 years.

### Intervention

From inception, RUHS’s CON program utilized a mastery-learning model and block system (Table 2). The 18-month, fulltime program includes a 2-week winter and a 1-week summer break. The program consists of 76.9 total credit hours defined as follows: didactic - 15 contact hours equal one credit hour; clinical lab - 30 contact hours equal 1 credit hour; and clinical experiences – that require 40 contact hours for each credit hour. During the didactic component of the curriculum, students focus on a single subject and must demonstrate mastery, defined as ≥90% on their assessment, to pass the block. If a student does not pass the block assessment, they receive feedback and a second opportunity to pass. Students who do not demonstrate mastery on repeat assessment are required to remediate during dedicated scheduled remediation sessions. The block format divides the material into smaller digestible elements which allows students to focus on a single subject area rather than juggling multiple classes. Block lengths are from 1 to 4 weeks long depending on the course and whether it includes a lab or clinical component. All blocks with the exception of the health assessment and nursing leadership blocks contain a didactic component, six blocks include lab training and simulation experiences and 8 blocks are experiential.

**Table:** **Table 2**

Block Number	Course Number	Course Title	Credits
1.0	300	Introduction to the Profession	3 Didactic
2.0	301	Health Assessment	3 Lab
3.0	302	Fundamentals of Nursing-Didactic	4 Didactic and Lab
3.1	302.1	Fundamentals of Nursing-Experiential	1.8 Clinical
4.0	303	Nursing Pharmacology	3 Didactic
5.0	304	Adult Health I-Didactic	7.5 Didactic and Lab
5.1	304.1	Adult Health I-Experiential	2.7 Clinical
		Remediation	
6.0	305	Nursing Theories, Practice and Practice Issues	3 Didactic
7.0	401	Nursing Research	3 Didactic
8.0	402	Maternal Newborn-Didactic	4 Didactic and Lab
8.1	402.1	Maternal Newborn-Experiential	2.7 Clinical
9.0	403	Pediatrics-Didactic	4 Didactic and Lab
9.1	403.1	Pediatrics-Experiential	2.7 Clinical
		Remediation	
10.0	306	Adult Health II-Didactic	7 Didactic and Lab
10.1	306.1	Adult Health II-Experiential	3.6 Clinical
11	409	Community Mental Health-Didactic	8 Didactic and La
11.1	409.1	Community Mental Health-Experiential	4 Clinical
		Remediation	
12	410	Care of the Older Adult	3 Didactic
		Remediation	
13	406	Nursing Leadership	3 Didactic
14	407	Senior Practicum	3.6 Clinical
		Remediation	
15	408	Senior Seminar	3 Didactic
Total Credits			76.9 Credits

For the didactic and skills portions of the curriculum, students are in session for 4 to 6 hours each day. The day combines traditional classroom instruction with active-learning including group studies and peer tutoring, programmed instructional units, and audiovisual games. Skill-based activities are incorporated into the curriculum to help students apply their knowledge into the clinical setting. The extended classroom day provides a platform for integration of didactic learning, active learning, and formative feedback which helps assure that students are progressing and mastering block content. Table 3 outlines a sample classroom day.

**Table 3:** Sample Classroom Day of The CON Mastery Model

Time	Classroom Schedule
8:00 to 8:45	Formative assessment “quiz” to identify any gaps in understanding from the previous day’s learning session. Students complete exercise as if they were in a summative assessment situation (no notes, no collaboration, etc.). Each item on the quizz is reviewed and discussed, much like an assessment review.
8:45 to 9:00	Break
9:00	Students are introduced to new concepts. Lecture with embedded
to	formative assessments/active learning. Breaks are taken
12:00	approximately every 45–60 min.
12:00 to 1:00	Lunch Break
1:00	Faculty either lecture for 1 h with 1 h of active learning or
to	faculty facilitate 2 h of active learning activities with in-class
3:00	review and discussion. Faculty circulates among teams to listen,
	assist, and ask question.

### Assessments

Assessments align with the block competencies and students need to achieve a score of ≥ 90% to pass an assessment. After finishing their individual assessment, each student joins a small team to retake the same assessment. If the team scores ≥90% on the exam, team members earn 5% points towards their own assessment score. The team process challenges students to defend their answers to their group, provides immediate feedback for students at the peer level, fosters teamwork and collaboration via shared decision making, and for disputed answers, affords students the opportunity to present a rationale for their answer and to defend it to the group. Stronger students benefit by teaching weaker students which reinforces concepts and critical thinking, while weaker students benefit from peer to peer learning. The team assessments also mimic clinical practice where health professionals may deliberate with colleagues about the best course of action. Following the team exam, instructors review assessments by explaining correct answers and providing a rationale for why other responses are incorrect. Reviews are interactive and students may challenge questions using an evidenced based response. Grades are pass or no pass. Students who receive a score ≥95% (including team points) receive an honors grade.

The RUHS model provides multiple opportunities for formative feedback to correct misconceptions and to reinforce concepts, making 90% passing a high but realistic standard. If students require remediation, instructors focus remediation sessions on identified deficiencies. If the material is not mastered after reassessment, students attend scheduled remediation sessions that are built into the curriculum.

Students who master the material on their first assessment have time to pursue enhancement activities, to conduct research, to work in a health related job, or to use as restorative personal time. Table 4 outlines how a mastery model differs from a traditional model with examples of how the Roseman model operationalizes the principles of competency based education.

**Table 4:** Comparison of Mastery Model, Traditional Model, and Roseman University Model

Mastery Model	Traditional Model	Roseman University Model
No grades – Students advance when they demonstrate mastery	Grades	Courses pass/fail
Focus on one subject at a time	Students have multiple subjects	Content broken down into digestible 2 week blocks focused on single subject
To pass must demonstrate mastery – no curve	Students are norm referenced, often graded on a curve	Must demonstrate mastery at 90% to pass
Less competition	Students may compete for best grades	No grades
Fosters teamwork with collaborative learning and group testing	Teamwork less emphasized, whole group instruction the norm	Team learning and team assessments that count toward an individual’s assessment points
Transparency empowers students	Students may be less certain about what it takes to pass	All blocks have clear objectives and to pass students need to demonstrate mastery of objective
Student instruction guided by frequent formative assessment	Little feedback before summative testing, instruction less tailored to individual needs	Multiple formative assessments with reviews. Identifies where students need help
Assessment is viewed as a positive learning experience	Testing may be threatening	Students know they have multiple chances to demonstrate mastery. Develops a culture where reassessment is not viewed as failure
Have multiple opportunities to demonstrate mastery	Single summative test may determine if student passed	Have multiple options to pass with focused reviews to address areas of deficiency
Students advance when the demonstrate mastery of material	Course often time based, advance at the end of a fixed period	Students who demonstrate mastery early on have opportunities for more advanced material, research or to enjoy personal time.

All nursing faculty receive training about teaching in a mastery-based learning model. Consistency across the curriculum is important since the model requires adherence to the curricular goals and competency measures developed by the CON. Faculty orientation includes learning about the RUHS model from an online educational module combined with a period of peer reviewed observation and mentoring by experienced faculty members.

### Design, Data collection and analysis

This is a descriptive study and used a retrospective analysis of data collected as part of the programs accreditation reporting and internal self-assessment to examine program outcomes. The CON admission’s office provided demographic data, graduation rates and attrition rates for all CON students enrolled in graduation classes from 2015 to 2017. De-identified student exit survey data was collected from the CON’s accreditation database. The data was uploaded to an Excel file and the Excel Statistical Analysis ToolPak was used to calculate descriptive statistics such as average age, GPA and TEAS averages. A t-test analysis was used to examine for differences in nursing license pass rates for RUHS students compared to national pass rates over the same time period. A chi-squared analysis was performed to examine for differences between campuses for student demographics and reported outcome measures.

### Ethics

The Institutional Review Board at Roseman University determined that the use of de-identified, retrospective accreditation database was not human research.

## Results

The RUHS CON completion rates from 2015 to 2017 ranged from 78.7% to 100%, exceeding the national standard of 70% or higher set by the Commission on Collegiate Nursing Education [23]. Employment for the combined cohorts exceeded the accreditation standard of at least 90% of those seeking employment at one year after graduation.

National pass rates for licensing exams ranged from 81.8% to 84.5% during the 2013-2017 periods. RUHS CON scores during the same period ranged from 82% to 100%. A two-sample t-test assuming unequal variances with an alpha of 0.05 was calculated, t (9) = 2.26 (*p* = .061), and found no difference between national and Roseman NCLEX pass rates. Table 5 summarizes data from a required student exit survey. The data shown are from 2015-2017 and represent the results since the CON merged its two programs. Of note is an overall satisfaction with RUHS’s CON educational experience of 4.38 (using a 1 strongly disagree to 5 strongly agree Likert scale) and an even higher rating of satisfaction with the block system of 4.74. Both the remediation process (4.32) and system of group testing (4.44) also received high marks. Students reported lower satisfaction with their educational preparation (3.47) and confidence in their problem-solving (3.33). The differences between campuses was not statistically significant.

**Table 5:** Roseman University College of Nursing Student Graduation Survey Exit Results 2015-2017^a^

Items Measured	Henderson Campus	South Jordan Campus	Combined
Respondents (n)	129	75	204
Percentage Response ^b^	89.0%	90.4%	89.5%
Overall, what was your satisfaction with---^c^?			
Your education at Roseman	4.48	4.20	4.38
The block-system of education	4.71	4.80	4.74
The system of group testing and collaborative learning	4.43	4.47	4.44
The system of remediation	4.34	4.29	4.32
Interaction with your program faculty	4.52	4.31	4.44
The program	4.29	4.15	4.24
Personal growth	3.49	3.23	3.40
Educational Preparation	3.54	3.35	3.47
Professional growth/Career	3.52	3.34	3.45
Problem solving	3.49	3.04	3.33
How well do you feel you were prepared to be---^c^?			
Competent professional	4.57	4.33	4.48
Caring professional	4.72	4.72	4.72
Ethical professional	4.69	4,68	4.69

^a^ Completed by graduating nursing students at the end of P3 year

^b^ Number returning survey divided by number of students graduating during 2015-2017

^c^ Likert-type scale with 1 = very unsatisfied to 5 = very satisfied

## Discussion

The complexity and uncertainties of the clinical environment can make developing clinical competence challenging for both students and teachers [24]. A traditional model that grades on a curve or sets passing at 70% may not be adequate to assure a health professional meets the skills and standards of their profession. In contrast, a competency model which demands that graduates demonstrate mastery in all aspects of a discipline’s core requirements seeks to eliminate inconsistencies where an excellent score in one area may balance a subpar performance and create gaps in the proficiencies expected for a nursing graduate. Hypothetically, mastery models minimize these gaps so that graduates meet the expectations of employers and the public who expect all nurses to be competent at what they do. Yet despite its theoretical appeal, few empirical studies explore the effectiveness competency based nursing education [25]. Most studies reporting on competency based education are limited by studying senior undergraduate nurses or post registration nurses undergoing specialized training [26]. Furthermore these studies tended to have small sample sizes and were often of poor quality. This paper adds to the literature by describing RUHS CON’s comprehensive, longitudinal curriculum based on the principles of mastery learning and by reporting on the program’s outcomes.

Our results indicate that RUHS CON outcomes data exceeds national standards for the National Counseling Licensure Exam (NCLEX) pass rates and graduation completion rates, demonstrating that a mastery model can be successfully implemented in an accelerated, comprehensive curriculum. The NCLEX is a standardized test used by all states in the US to determine if a candidate is ready for licensure as a nurse. Exceeding national standards for pass rates on the NCLEX is an important external marker for validating the quality of a nursing program [27]. While to our knowledge this is the first paper reporting on a comprehensive CBE undergraduate program, positive outcomes were previously reported by an online RN to BSN program using a competency based learning model [28]. Similarly, a systematic review of competency based education of nursing students found that five of six studies demonstrated benefits from an outcomes based curriculum. Results from other health professions also suggest that competency based models can enhance training [29–31]. While limited, the existing evidence for CBE supports the results reported here that a curriculum built around mastery learning can be successful.

Another key finding is that students reported a high degree of satisfaction with the model. Students also reported favorably on the block system which breaks material into more manageable “bite-sized” segments which allows students to focus on one topic at a time. While student approval of frequent testing seems counterintuitive, students reported a high degree of satisfaction with the program’s assessment and remediation process suggesting that students may value constructive feedback aimed at improvement over simply earning a “grade”. This may also be reflected in the students’ rating the learning environment as positive. In addition, the curriculum yields graduates who report confidence in their ability to work as nurses.

### Limitations

There are several limitations to our findings and challenges that others considering a mastery model should consider. First, it is important to note that RUHS is a single institution founded on the premise that competency-based education is best for health professionals. The model might not translate into settings where the leadership does not fully embrace this perspective. Additionally, our analysis is primarily descriptive. We compare our outcomes with national standards and national averages wherever possible, but we do not have a matched comparator to use as a true control. A strength is that this paper adds to the literature by being the first to describe outcomes related to a comprehensive longitudinal competency based nursing program. It also reports on three years of data over two campuses. The ability to employ the curriculum in two distinct locations also suggests that the model can be applied to different settings.The assessment process, while not a limitation per se, does present a potential challenge. A commitment to a mastery model requires that the assessment process be robust, multifaceted, and tie back to the established criterion. For programs considering CBE, the assessment process for both the formative and summative evaluation can be a daunting challenge to implement [32]. Another concern is that by boiling content down to multiple bite-sized curricular elements, the art and complexity of real world nursing might be lost, especially in terms of professional identity and ethical behavior. However, by self-report, RUHS students express a high degree of confidence in their preparation to be both caring and ethical professionals. One area students reported slightly lower confidence in is problem-solving. This suggests that programs need to be careful that higher order competencies such as problem-solving are defined and assessed and not lost in the process of breaking nursing competencies into smaller components.

Another challenge to consider is that faculty need to devote sufficient time to feedback, deliberative practice, and other key components of mastery learning. Most faculty members either taught in, or received their training in, a traditional model and may not always embrace the mastery model. To be successful, a mastery model requires an investment in training new faculty and reinforcing the model to existing faculty. Another issue is that anecdotally faculty report block assessment reviews sometimes degenerate into students arguing for points, limiting the utility of assessment reviews as a learning experience. This may be a consequence of setting the bar at 90% where students scoring near that level may fiercely argue for extra points to avoid remediation.

A limitation to a mastery model is that transcripts do not generate a GPA. Even though a competency based program might provide a detailed explanation about how students need to achieve mastery, some graduate nursing programs in the United States use GPA as a criterion for admission and graduates seeking advanced nursing training may find the lack of a GPA is a barrier to admission. As competency based systems become more prevalent this may be less of an issue, but currently remains a limitation for schools to consider when implementing a mastery model.

Finally, our reported outcomes do not include a financial analysis and a mastery learning model might be costlier both for institutions and students. For example, Leung suggested that competency based programs adds more administrative cost compared to a traditional program [22]. However, Roseman tuition and staffing is comparable to other non-profit, private institutions implying that costs are similar to comparable institutions.

## Conclusions

The RUHS model illustrates that a comprehensive nursing school curriculum can be developed using a mastery-based learning model and lead to positive outcomes in terms of licensing pass rates, acceptable attrition rates, and broadly favorable student perceptions of their educational experience. The program’s success suggests that other nursing programs might be able to develop a curriculum based on a mastery based learning model. Further research is needed to explore how this model translates into other settings and how masterly learning compares to more traditional models.

## Data Availability

The datasets generated and/or analyzed during the current study are not publicly available as they are internal surveys graduating student surveys but are available from the corresponding author on reasonable request.

## Abbreviations

AACN: American Association of Colleges of Nursing
CBE: Competency-based education
CON: College of Nursing
NCLEX: National Council Licensure Examination
RUHS: Roseman University of Health Sciences
TEAS: Test of Essential Academic Skills
